# The Abuse of Medical Charities

**Published:** 1877-12

**Authors:** Orville F. Rogers


### MISCELLANEOUS.

### THE ABUSE OE MEDICAL CHARITIES.*

BY ORVILLE F. ROGERS, M. D.

An examination of the reports for last year of all the larger medical charities of the city shows that 92,977 patients were treated

* Read before the Norfoik District Medical Society.

gratuitously in the dispensaries and out-patient departments of the hospitals. The reports of the out-patient departments of the institutions which were in existence ten years ago show that the number treated by them during the past year was about three hundred per cent. greater than in 1866. Not only has the number of beneficiaries of the older institutions increased at this extraordinarily rapid rate, but new charities have been founded and well advertised, and are now, in the language of the reports, doing a "great work." The result of this is that more than twenty-seven per cent. of the population of Boston are treated by the dispensaries and out-patient departments of hospitals. This is an increase far out of proportion to the growth in population or the increase in the number of those relieved by other departments of the various charities. These facts are calculated to produce the impression that the number of medical paupers is greater than it should be, and that there is something wrong in the system of medical relief under which such a state of things can exist. An examination of the mode of administration which obtains at present tends to confirm this impression.

Before admitting a patient to a hospital the authorities satisfy themselves by a critical examination of all the circumstances of the case that the applicant is a person upon whom charity may be properly bestowed. The character of a certain portion of every community renders this course necessary if the charitable contributions of the public are not to be squandered upon professional vagabonds, or that slightly more respectable class who, though able to support themselves if obliged to do so, have neither sufficient self-respect nor honesty to restrain them from availing themselves of every easily obtained charity. Though all this is well known, I have been unable to learn that any efficient restrictive supervision of the out-patient departments and dispensaries is exercised by the managers of these institutions in Boston. Practically they are open to all. When a charitable institution is administered in such a manner as to *invite* fraud, it is not strange that its consultation rooms are thronged with crowds of shameless imposters as well as by the deserving poor. In view of all the opportunities there are for obtaining gratuitous medical advice, it is surprising that so many of the poorer classes still employ and pay a doctor. Ignorance of the privileges offered or self-respect have heretofore kept many from becoming hangers-on of the out-patient department; but the ignorant are rapidly becoming informed and accept what is so freely given, and the scruples of the would-be honest are overcome by the influences of the out-patient consultation room, a place where medical advice is so cheapened that it is a question whether patient or doctor confer the favor, where there is no official to ask unpleasant questions as to the pecuniary ability of the applicant, and where a cheerful doctor sits with somewhat of the air of a man giving a reception to his friends, and bids them all as they depart to "come again." The cry of "hard times," while properly raised by some, offers a ready-made excuse to those who have just honesty enough to feel the need of a salve for their consciences while they are stealing an ointment for their shins. It is not claimed that many of the really well-to-do class are found here, but the unscrupulous cupidity of some leads them to don their uniform of old clothes and enlist for a limited term of service in the ignoble army of medical "bummers."

It is impossible at present to say what proportion of those who apply for gratuitous treatment are able to pay. At the Massachusetts General Hospital the voluntary contributions of out-patients during 1876 was one hundred and twenty-six dollars; but this sum cannot be supposed to represent the paying capacity of its seventeen thousand out-patients. It undoubtedly represents several thousands of dollars which these people would have paid the profession as fees for services had not the hospital gratuitously furnished them.

It was found at the London Hospital, Whitechapel Road, that forty-nine per cent. of the out-patients should not have applied there. Probably a similar state of things exists in this country. Whether the proportion of imposters is as large in Boston as in London is immaterial to the discussion, as the one point to be emphasized here is that the out-patient and dispensary systems *invite* patients and frauds instead of raising suitable safeguards in the interests of justice. In order that one acquainted with the system may suppose that grave abuses do not exist, he would have to believe that the mass of the lower orders of the community are possessed of incorruptible honesty, of self-respect and a nice sense of honor, which propositions will hardly be accepted by one who has practiced medicine for any length of time.

The blame for the present condition of affairs largely rests with the members of the medical profession. If physicians were not willing and even anxious to give their services in the out-patient department for the sake of clinical advantages, this system would be speedily overthrown. So long as the cost of maintaining hospitals and dispensaries is not materially increased by wholesale charities of this character, the public cannot be expected to interest itself in the matter; but if it were to cost the institutions a small sum for each patient, checks upon this abuse would be immediately applied. Hence those who serve gratuitously in the out-patient departments and dispensaries are mainly responsible for the existing system. These men cannot claim that they do this solely in the interest of suffering humanity, for the ends they have in view are chiefly selfish. They hope through the many advantages of the place to gain a reputation and a lucrative practice, and to fit themselves to fill prominent positions in the profession. Whether these objects, worthy in themselves, justify a few men in disregarding the interests of the profession at large is a question to which there can be but one reply. It is an unpleasant fact that the new dispensaries and departments of out-door relief devoted to the various specialties are usually opened fully as much for the advantage of those who are to have charge of them as for the suffering poor, and every effort is made to justify their establishment by extending their benefits to nearly every one, worthy or unworthy, who may apply at their doors, and upon the fact that large numbers are willing to accept this aid is based an appeal to the public to contribute generously, in order that this so-called "beneficent work" may be prosecuted on a still greater scale.

There is a growing feeling in the profession that this system is wrong, and that a reform is needed. Unfortunately, this feeling is not shared by the medical officers of these charitable institutions, who are rather inclined to resent any attempt to diminish the number of patients as an assault upon their privileges. It is not easy to find a remedy for the evils complained of.

In England, where the subject has excited considerable interest, the provident dispensary plan has been tried with a certain measure of success. An effort has been made to establish a similar institution in New York, but so far it has been without any tangible result. This is not surprising, since the provident dispensary, based upon the just principle that valuable services demand reasonable payment, is obliged to compete in the same field with old institutions which gratuitously render the same services. It has been proposed to charge all applicants at the out-patient department a small fee, but this is obviously inexpedient, and so far as I can learn has not been tried. Certainly no more efficient plan could be devised for defeating the very end for which the out-patient department was established, namely, the relief of the destitute * The New York Hospital has adopted the system of selling the privileges of the out-patient department for a dollar a month to any who may apply. These plans are wrong in principle, and the profession should earnestly protest against their further adoption. The dollar-a-month system, especially, is an innovation so dangerous to the interests of the profession and so manifestly wrong that it is astonishing that reputable physicians are willing to assist in carrying it into execution. If this movement is widely imitated it will not be long before the profession will have nearly all the indigent sick upon its hands, and the hospitals will have the bulk of the paying patients of the poorer class. If this plan has been devised as a remedy for present ills, it is a striking example of a remedy that is worse than the disease. It has been thought that if the number of physicians to out-patients were increased, and they were instructed to institute a searching inquiry into the pecuniary ability

* Since writing the above I have learned that the Massachusetts General Hospital has adopted this plan of demanding a fee from all, and that they make some inquiry into the circumstances of those who claim to be unable to pay. It is to be hoped that the hospital will ultimately turn away those able to pay a fee and attend only those who are its legitimate patients of all applicants, and to send away those able to pay a moderate fee, the evil would be soon overcome. This method would undoubtedly succeed if the cordial co-operation of the medical officers could be secured; but this, for reasons before given, cannot be expected. If this duty were entrusted to an officer, not a medical man, who should carry his inquiries into the homes of all who became patients, imposition on any large scale would be prevented.

The proposal to abolish the out-patient department has been adopted by the managers of two hospitals in London, who were unable otherwise to rid themselves of the horde of barefaced impostors that infested their consultation rooms. The Board of Jewish Guardians of London has also taken similar action. Their decision in this matter is worthy of consideration, since the Jews are acknowledged to be most skillful and humane in the care of the poor. The only valid objection to this course that can be raised is that the sick poor would not be cared for. There is little probability that any case would go untreated, since humanity and self-interest alike prompt the profession to render aid whenever it is required. The abolition of the out-patient department would throw a large burden upon the profession, but the greater part of it would fall upon the young practitioners, who would gladly assume it for the sake of the experience and the moderate pecuniary recompense to be gained. Perhaps the radical expedient of abolishing the out-patient department may not be adopted in Boston; certainly it will not be if that large and influential body of men connected with the medical schools and the hospitals can help it. Nevertheless, some plan ought to be devised to prevent the shameless begging which now prevails. It will be, and the evil system, fostered by the active connivance of a portion of the profession and maintained by the conservatism and indifference of the managers of medical charities, will be overthrown.

One of the worst features of the wholesale relief system is that the loss falls heaviest upon those who can least afford to bear it,—the younger members of the profession. During the earlier portion of the professional life of most men the poor are their best friends and their only patients. In fact, the poor are the very breath of their nostrils. Even a patient unable to pay a penny is often of much benefit to his doctor. It is needless to dilate upon this point, as every physician knows that the poor offer the young man almost his only field for making a living and a reputation. A large portion of that field has already been taken from him, and if the present order of things is to continue and the number of medical beneficiaries is to increase during the next decade as it has in the last, not only will the portion of the young man be lost, but some of the older brethren will have to say with the Psalmist: "Thou broughtest *us* into the net; thou laidest affliction upon *our* loins."—*Boston Medical and Surgical Journal.*

